# Temporally dependent pollinator competition and facilitation with mass flowering crops affects yield in co-blooming crops

**DOI:** 10.1038/srep45296

**Published:** 2017-03-27

**Authors:** Heather Grab, Eleanor J. Blitzer, Bryan Danforth, Greg Loeb, Katja Poveda

**Affiliations:** 1 Department of Entomology, Cornell University, Ithaca, New York 14853, United States; 2 Department of Entomology, New York State Agricultural Experiment Station, Cornell University, Geneva, New York 14456, United States; 3 Department of Biology, Carroll College, 295 S. Harrison Helena, MT 56901, USA

## Abstract

One of the greatest challenges in sustainable agricultural production is managing ecosystem services, such as pollination, in ways that maximize crop yields. Most efforts to increase services by wild pollinators focus on management of natural habitats surrounding farms or non-crop habitats within farms. However, mass flowering crops create resource pulses that may be important determinants of pollinator dynamics. Mass bloom attracts pollinators and it is unclear how this affects the pollination and yields of other co-blooming crops. We investigated the effects of mass flowering apple on the pollinator community and yield of co-blooming strawberry on farms spanning a gradient in cover of apple orchards in the landscape. The effect of mass flowering apple on strawberry was dependent on the stage of apple bloom. During early and peak apple bloom, pollinator abundance and yield were reduced in landscapes with high cover of apple orchards. Following peak apple bloom, pollinator abundance was greater on farms with high apple cover and corresponded with increased yields on these farms. Spatial and temporal overlap between mass flowering and co-blooming crops alters the strength and direction of these dynamics and suggests that yields can be optimized by designing agricultural systems that avoid competition while maximizing facilitation.

Increasing consumption driven by a growing global population and demands for more varied and resource intensive diets has placed unparalleled strain on our agricultural production systems and natural resources. Current agricultural practices rely on fossil fuels, agrochemicals and conversion of new agricultural lands. Yet, yield gains produced through these practices have plateaued in recent years^1^ and have come at the cost of increasing greenhouse gas emissions, degradation of water quality, widespread pollution, pesticide resistance and unprecedented biodiversity loss. An alternative solution to meet the planet’s growing needs is ecological intensification, increasing production on existing farmlands in ways that causes less harm on the environment through the replacement of anthropogenic inputs with ecosystem services management^2^,^3^. Manipulating and regulating the biotic interactions underpinning the provisioning of ecosystem services by increasing the structural diversity of agroecosystems had been demonstrated to improve crop yields^4^,^5^,^6^. In order to implement diversification strategies successfully, it is critical to understand whether agricultural habitats themselves may act as sources of ecosystem services or whether diversification may lead to competition for services among crops. Certain crops may have a disproportionate effect on the flow of ecosystem services due to the large pulse of resources they provide^7^,^8^, and it is essential to understand the effects of these crops on ecosystem service dynamics in order to develop effective management strategies that can be directly implemented by land managers.

Crops that are grown on large scales and bloom *en masse* create large pulses of resources that have substantial impacts on communities of ecosystem service providers. These dynamics are particularly relevant for pollinator dependent crops given the dramatic increase in the area planted to these crops^9^ and their importance for human nutrition^10^,^11^. Pulses in floral resources associated with mass blooming of crops are known to alter pollinator abundances and visitation rates in nearby crops and natural habitats^12^,^13^,^14^,^15^, which are likely to have direct impacts on crop yields^16^. Mass flowering crops can increase pollinator offspring production^17^ and pollinator densities following mass bloom^18^,^19^, particularly for solitary, univoltine bees^20^ for which the bloom of a single crop may represent nearly the entire span of their adult foraging activity. Therefore, mass flowering crops may facilitate pollination of co-blooming crops when pollinators attracted and supported by the mass blooming crop spill over into the co-blooming crop, augmenting floral visitation and crop pollination^21^ (Fig. 1). Alternatively, when pollinators are limited, as is common in simplified agricultural systems^22^, plants with high overlap in their pollinator community may compete for visits from shared pollinators^23^ (Fig. 1).

Little is known about competitive or facilitative interactions between pollinator dependent crops with respect to pollinators and pollinator services; however, we expect that these interactions are ubiquitous in agricultural landscapes. They only require that crops have overlap in their pollinator community though they may bloom in different seasons^21^ or even in different years^20^. The likelihood of the interaction resulting in either facilitation or competition depends not only on the degree of overlap in the pollinator community^15^ but also on the temporal overlap in bloom between crops^13^,^21^,^24^. Indeed, the two studies available on the effects of mass flowering crops on wild plants have found that mass flowering crops can either reduce^25^ or enhance pollination^13^ in co-blooming plants in nearby natural habitats.

At the landscape scale, greater abundance and diversity of bees associated with natural and semi-natural habitats^22^ may buffer against local competition or facilitation effects. For example, in landscapes with high amounts of natural habitat, competition between co-blooming crops may be lower than expected^14^. In this case, although bees are drawn to the mass blooming crop, the number of bees moving from natural habitats into the co-blooming crop may still be sufficient to provide adequate pollination services^7^. Alternatively, proximity to natural habitats may reduce facilitation when bees move from mass blooming crops to alternative forage in natural habitats rather than the co-blooming crop.

Despite the potential importance of pulsed resource dynamics for crop pollination and associated yield, we are not aware of any studies that have evaluated the effects of mass flowering crops on the yield of another crop. Greater understanding of spatial and temporal factors that shift the balance between competition and facilitation will allow for management practices that maximize crop yields under the pulsed resource dynamics characteristic of agroecosystems.

In this study we investigate the potential for pollinator mediated competition or facilitation in two economically important crops: apple (*Malus domestica*), a mass flowering crop, and strawberry (*Fragaria x ananassa* Duch.) in central New York, USA. In this region apple and strawberry have a staggered but overlapping bloom period. The two crops are both members of the family Rosacea, and thus expected to have high overlap in their pollinator faunas^26^,^27^. Furthermore, the community of bees visiting both apple and strawberry is dominated by early spring, ground-nesting, univoltine bees in the genus *Andrena*^28^,^29^,^30^. The high potential for community overlap in pollinators between apple and strawberry make these two crops an ideal study system in which to understand the potential for pollinator-mediated interactions among crops.

We hypothesized that the impact of apple on strawberry pollination may vary temporally, with facilitation and competition occurring in the same system but at different stages of apple bloom. Additionally, we hypothesized that proximity to natural habitats would moderate these effects and predicted that sites in close proximity to natural habitat would have greater bee abundance and experience both reduced competitive and facilitative effects.

## Methods

Both apple and strawberry are economically important crops in the United States, with total apple production at 327,000 acres and strawberry production at 61,000 acres (USDA NASS, 2013). In New York State, the second largest apple-producing region in the US (USDA NASS, 2013), it is common for farms to grow apples plus a range of other small fruit crops including strawberry.

### Study Sites

Studies were carried out in the spring of 2013 in the Finger Lakes Region (42°26′N, 76°30′W) of New York, USA. The study area is characterized by a diversity of agricultural uses, including dairy, row crop, tree fruits and vegetables with natural and semi-natural areas of deciduous forest, small woodlots and old field dispersed throughout. A total of 35 farms growing apple, strawberry or both were identified. All farms were used to estimate pollinator community similarity and a subset of 13 farms, all growing strawberry but with a gradient in the proportion of apple orchard cover in the surrounding landscape (0–37%), were used in further experiments. Focal strawberry fields on each of these 13 farms were selected. The landscape surrounding the focal strawberry field was characterized within a 1 km radius using the 2013 National Agricultural Statistics Service Cropland Data Layer for New York^31^ in ArcGIS 10.1. Using these maps we estimated the proportion of land in agricultural uses (all crop categories including forage and pasture), natural and semi-natural areas (forest, wetlands, shrub lands, meadows, and fallow), and apple orchards. Apple orchards flowered between May 3 and June 5, 2013, with bloom initiation and duration varying across farms depending on local microclimate and apple variety. Most farms cultivate a number of apple varieties to ensure pollination success, as apple is varietally self-incompatible. Measurements occurred between May 6 and May 9 for early apple bloom, between May 14 and May 17 for peak apple bloom and between May 31 and June 3 for late apple bloom. Apple flowering phenology in 2013 would be described as “typical” based on historical data on apple flowering in upstate NY^32^. In the early stage of apple bloom the percentage flowers open of total flowers present, estimated by counting the number of open flowers per cluster on randomly selected trees, averaged 26.6% (±5.4 SE). During the same period, strawberry bloom had initiated only at four sites (with 16.9% ± 11.5 SE flowers open). At peak apple bloom, flowering intensity averaged 54.8% (±5.8 SE) compared to strawberry bloom at 23.2% (±7.3 SE). Apple bloom intensity during the late flowering stage averaged only 10.9% (±3.5 SE) while strawberry bloom was 37.5% (±6.7 SE).

We quantified apple mass flowering using a mass flowering index. The index describes the total amount of apple flowering within the surrounding landscape and is calculated as the percent apple cover in a 1 km radius around the focal strawberry field multiplied by the percent of open apple flowers in adjacent orchards (if present). Thus, the highest values of the mass flowering index indicate high abundance of apple flowers locally and within the landscape.

### Pollinator Community

To estimate similarity in the apple and strawberry pollinator communities, bees were collected using sweep netting during four 15-minute surveys along 100 m transects in apple orchards and strawberry fields during the peak bloom of each crop. Surveys were conducted between 10:00 and 15:30 on sunny days with temperature above 16 °C. Bees were identified to species using published revisions^33^,^34^,^35^,^36^,^37^,^38^ and online keys (Discoverlife.org) as well as expertly identified reference materials maintained in the Cornell University Insect Collection (http://cuic.entomology.cornell.edu/).

In order to understand how mass flowering apple impacts bee visitation to strawberry, we estimated the abundance and diversity of bees visiting strawberries over the course of the apple bloom within each focal strawberry field and adjacent to the nearest natural or semi-natural habitats. Distances between strawberry fields and semi-natural habitats on a farm ranged from 20 to 300 m (mean = 46 ± 9 m). Arrays of four white pan traps were placed at 3 m intervals on transects 2 m from the edge of each focal strawberry field and semi-natural habitat during three sampling periods corresponding to the early, peak and late stages of apple bloom. White pan traps were used as they collect a greater number of bees but maintain a similar community composition to sweep net sampling compared to other trap colors (H. Grab, *unpublished data*). Sampling periods were approximately one week apart, varying based on local microclimatic conditions, beginning on May 6^th^ and ending June 3^rd^ 2013. The intensity of strawberry and apple bloom was recorded when the arrays were set out and when they were collected. Bloom intensity was estimated as the percentage flowers open of total flowers present including senesced flowers and flowers in bud stage in the orchards or fields. These data were then averaged in order to estimate bloom intensity during each stage. Pan traps were collected after 72 hours and the contents were sorted and identified to species. Sampling rarefaction curves for species richness are available in the Supplementary Information (Figs S1–3).

### Strawberry pollination

To assess the effects of apple mass flowering at the landscape scale on strawberry pollination and fruit set, we measured the pollination rates of sentinel strawberry plants placed within the focal strawberry field and adjacent to semi-natural habitat at each farm. Use of sentinel plants allowed us to maintain consistent strawberry bloom density during each stage of apple bloom and to control for abiotic factors, including soil and microclimate that could affect yield. During the three periods corresponding to the early, peak and late stages of apple bloom, we placed 10 individually potted strawberry plants (variety Evie 2) in the same transects used for pollinator sampling described above. Strawberry plants have one primary flower, two secondary flowers and up to four tertiary flowers per inflorescence. The number of achenes is greatest on primary fruit and decreases in subsequent flowers. Only primary and secondary fruits were used to estimate yield, as they are the only fruits usually considered marketable. These flowers were exposed to visitors for 72 hours but on half of the plants, flowers received supplementary pollen applied with a paintbrush to the stigmas. These hand-pollinated fruits, when compared to open-pollinated fruits, allowed us to estimate the relative contribution of the pollinator community to yield while controlling for environmental factors such as microclimate, which may have varied slightly across the study region. We collected the sentinel plants after 72 hours and maintained them in a greenhouse chamber while the fruits developed. Fruits were harvested daily when ripe and weighed. In strawberries, fruit weight can provide an accurate estimate of pollination rate, as strawberries are an aggregate accessory fruit comprised of as many as 300 individual achenes^39^. Each achene must be fertilized in order for the surrounding tissue to develop. Hence, the weight of a fruit is highly correlated with the number of pollinated achenes^40^ and an average of four pollinator visits per flower is required to achieve full pollination and maximum fruit weight^41^. Only fruits with a high percentage of fertilized achenes will develop without major malformations that reduce overall yield and marketability.

### Statistical Analyses

The effect of apple mass flowering on bees was assessed using mixed effects models in the R package “nlme”^42^ with either the dependent variable of bee abundance (total number of bees collected during a sampling stage at each site) or bee diversity (number of bee species collected during a sampling stage at each site) in separate models. In both models the fixed effects included natural habitat proximity (adjacent or distant from nearest natural area), apple mass flowering index, the percentage of strawberry bloom, apple bloom stage (early, peak, or late), and all possible interactions between the fixed effects. Mass flowering index was log(x + 1) transformed to meet distributional assumptions. Farm was included as a random effect in the model describing bee abundance. In the final model describing bee diversity, natural habitat proximity nested within farm was included as a random effect because proximity was removed as a fixed effect following model simplification.

We used linear mixed effects models to assess the relationship between bee abundance and diversity and the average weight of strawberry fruits. Models were fit separately for bee abundance and diversity as fixed effects along with pollination treatment, apple bloom stage and location. Following model simplification the final models retained only the main effects of abundance or diversity. To account for non-independence of samples and the nested experimental design structure, random effects in the final model included the nested effects of stage within pollination treatment within the natural habitat proximity variable within farm.

In order to determine the indirect effects of the apple mass flowering index on strawberry fruit weight during each of the apple bloom stages, we used separate mixed effects models with fruit weight as the response variable. The predictor variables included pollination treatment, the mass flowering index, and all possible interactions between the fixed effects. Fruit order (primary or secondary) nested within the natural habitat proximity variable nested within farm was included as a random effect in each model to account for the nested sampling design. In the model describing the effects during peak bloom, weight was log transformed to meet distributional normality assumptions.

All models were computed in R v. 3.2.3^43^. Minimum adequate models were selected using backwards-stepwise selection, eliminating predictor variable with p values < 0.1. Once minimum adequate models were identified, the anova function was used to assess significance of each factor by obtaining *F* and *p* values. In all models apple mass flowering index values were log_10_(x + 1) transformed to account for overdispersion due to some farms having very high percentages of apple cover.

## Results

### Community Similarity

**Figure 2:**
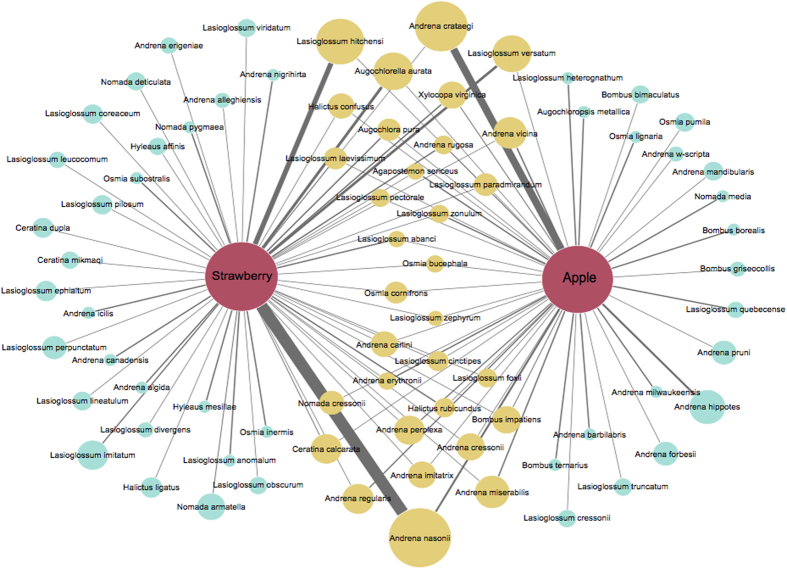
Pollinator communities of apple and strawberry in the Finger Lakes region of New York State. Node size indicates total abundance and edge widths represent relative abundance in each crop. Yellow = shared, Blue = unshared.

Using bees collected in sweep-net transects in apple orchards (*n* = 18 orchards, abundance = 776, species = 51) and strawberry fields (*n* = 17 fields, abundance = 994, species = 60) during peak bloom of each crop, we compared the overlap in pollinator communities of each crop. We found that apple and strawberry share 31 of the 79 pollinator species collected including the most abundant pollinators of each (Fig. 2). In this region, honey bees, *Apis mellifera*, are often brought into orchards for apple pollination but not for strawberry. These managed honey bee colonies are moved out of apple orchards following peak apple bloom; therefore, we present honey bee abundance separately from the wild pollinator community. In apple orchards, honey bees comprised 48% of the pollinator community; while in strawberry, honey bees comprised only 1.3% of the bees collected. Because our estimates of community overlap are based on collections from geographically separated locations, they represent a conservative measure of the overlap in apple and strawberry pollinators that is likely to occur within a farm.

### Bee Response to Mass Flowering Apple

**Figure 3:**
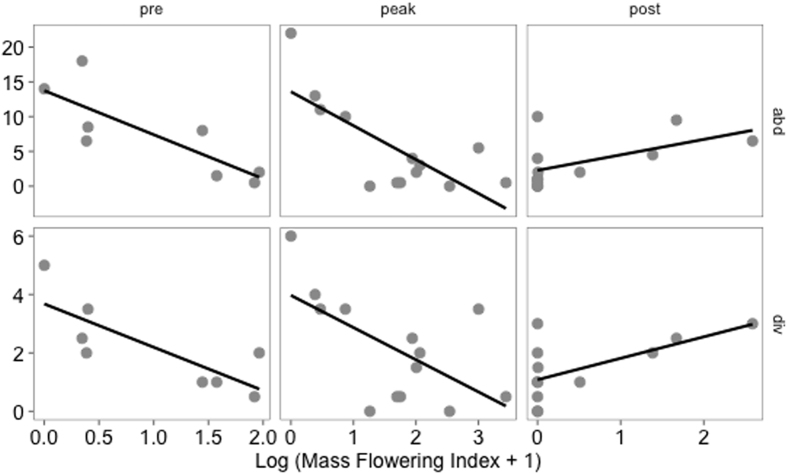
Average bee abundance and species richness during early, peak and late apple bloom in relation to the mass flowering index which describes the total amount of apple flowering within the surrounding landscape.

There was a significant effect of apple mass flowering on the abundance and diversity of bees collected in strawberry fields and adjacent semi-natural habitats that was dependent on the bloom stage (Table 1 and Fig. 3). When further exploring the interaction between stage and the mass flowering index (Table 1) we found that abundance and diversity of bees collected near the sentinel plants were negatively affected by mass flowering during both early and peak apple bloom and positively affected by mass flowering during late apple bloom (Fig. 3, Table S1). Bee community composition was marginally effected by the stage of apple bloom (Figure S4). As expected, bee abundance was marginally higher adjacent to semi-natural areas (mean = 16.14, SE = 2.47) compared to strawberry fields with no adjacent semi-natural habitats (mean = 9.01, SE = 2.21 Table 1). However, natural habitat proximity did not interact with either stage or the mass flowering index suggesting that the proximity to natural habitat did not alter the impact of mass flowering on the pollinator community. Although species richness was not different between strawberry fields and natural habitats, the composition of bee communities differed between locations (Figure S5). The local intensity of strawberry bloom did not impact bee abundance or diversity at any stage, and was therefore removed from all models.

### Strawberry Yield

**Figure 4:**
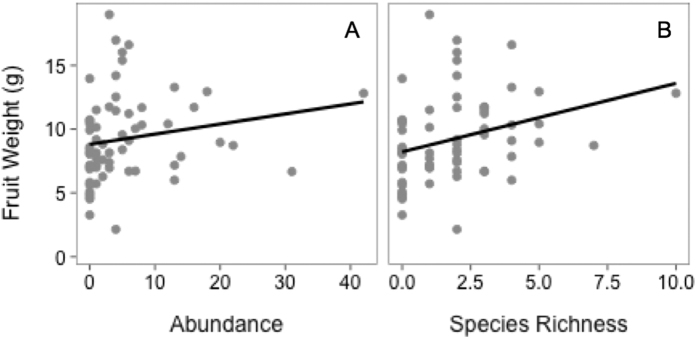
Averaged weight of strawberry fruits per farm relative to (**A**) bee abundance (**B**) bee species richness. Regression lines indicate significant relationships (p < 0.05).

**Figure 5:**
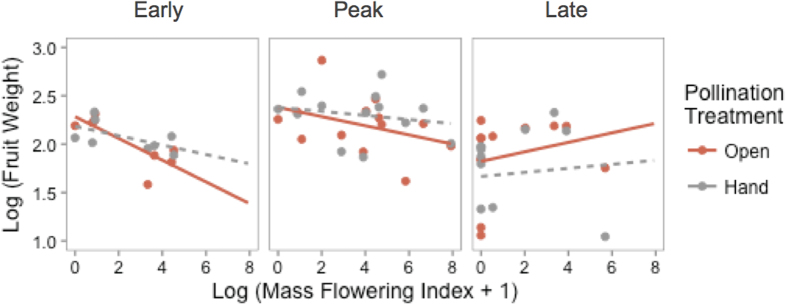
Average weight of hand-pollinated and open-pollinated strawberry fruits during early, peak and late apple bloom relative to the mass flowering index (calculated as the percentage of apple in the landscape multiplied by the intensity of apple bloom for each sampling period). Regression lines indicate significant relationships (p < 0.05).

The average weight of strawberry fruits from sentinel plants increased with both greater bee abundance (*F*_1,13_ = 5.72 p = 0.03) and diversity (*F*_1,13_ = 24.22 p =  <0.001) (Fig. 4). Although pollinator abundances were greater near to natural habitats, fruit yield did not vary with natural habitat proximity. Similar to the effects observed on the pollinator community, we found the effects of apple mass flowering on strawberry fruit weight differed with the stage of apple bloom (Table 2). During both early and peak apple bloom, an interaction between pollination treatment and mass flowering impacted strawberry fruit weight (Table 2). In accordance with the competition hypothesis, mass flowering of apple decreased the weight of open pollinated strawberry fruits but not hand pollinated fruits (Fig. 5, Table S2). Conversely, during late apple bloom the mass flowering index was positively associated with fruit weight (Fig. 5, Table S2) suggesting facilitation during this stage.

## Discussion

Resource pulses are a common feature of agricultural systems; however, the impact of mass flowering crops on the pollination and yield of co-blooming crops is currently unknown. Here we examined the spatial and temporal effects of a mass flowering crop on bee communities and subsequently on yield in a co-blooming crop species. We predicted that changes in pollinator abundance over the course of mass flowering would lead to either competition or facilitation at different stages, and indeed we found that apple mass flowering first decreased strawberry pollination and then increased strawberry pollination with corresponding effects on yield.

The mass flowering of apple negatively affected bee abundance and diversity in co-blooming strawberry during the early and peak stage of apple bloom. However, during the late bloom stage, increasing apple mass flowering was associated with greater bee abundance and diversity in strawberry. These results indicate that bees are responding to local changes in resource availability resulting in a dilution of bees when floral resources are plentiful during early and peak apple bloom followed by a spillover of bees from apple orchards to nearby strawberry fields as apple flowering decreases. In natural systems, similar effects have been observed in mixtures of flowering *Cirsium* and *Raphanus* plants where the balance between pollinator mediated competition and facilitation was dependent on the relative densities of *Cirsium* flowers^44^. These patterns may be explained by changes in the foraging preferences of the pollinator community, but population level responses to floral resources pulses may support overall greater abundances of pollinators in landscapes with high cover of mass blooming crops^18^,^19^,^21^. Our findings indicate that both density and timing of flowering are important predictors of the outcome of these interactions.

We predicted that both abundance and diversity of bees would be greater at sites adjacent to natural habitats. Although bee abundance was greater at sites adjacent to natural habitats, bee species richness did not differ between sampling locations. This result is likely due to a greater density of nesting sites in less disturbed natural areas for the ground-nesting bees that dominated the pollinator community^45^. While the distance between strawberry fields and semi-natural habitats within a farm was not greater than the flight distance of the average strawberry pollinator^46^, it is possible that fewer individuals traveled that distance. Despite overall greater pollinator abundances in sites adjacent to natural habitats, lack of a significant interaction between the natural habitat proximity variable and mass flowering indicated that proximity to natural habitat did not alter the impact of mass flowering on the pollinator community. Furthermore, the influence of mass flowering apple on the abundance and diversity of bees was greater than the influence of proximity to natural habitats. Similar results were reported by Westphal *et al*.^18^, who found that bumble bee densities were positively related to the availability of oilseed rape and not natural habitats within the landscape. Our findings reveal that these effects extend to a much broader pollinator community. These findings also suggest that the effects of agricultural habitats on pollinator communities has thus far been underestimated and likely represents a common phenomenon among crops with overlapping pollinator communities.

Mass flowering of apple at the landscape scale was negatively associated with the weight of open pollinated strawberry fruits during early and peak apple bloom and positively associated with fruit weight during late apple bloom. We hypothesize that these results are due to the parallel changes observed in the abundance and diversity of pollinators, as both measures were highly correlated with the weight of open pollinated strawberry fruit; however, the decrease in fruit weight associated with early and peak apple bloom may also be due in part to increased rates of heterospecific pollen transfer^47^ from apple to strawberry. In the late sampling period, the positive response of hand-pollinated fruit to the mass flowering index may have been caused by incomplete effectiveness of the hand pollination treatment due to the greater storage time of the supplemental pollen at this stage.

The competitive interactions observed between apple and strawberry likely represent a conservative estimate of the potential magnitude of indirect interactions mediated by shared pollinators. In this case, competitive effects are moderated by the relatively diverse pollinator community of strawberry^48^ and the ability of strawberry to self-pollinate^41^. Therefore, the negative effects of mass flowering may be stronger in crops that are more pollinator dependent or share a greater proportion of their pollinator community with a mass flowering crop.

In natural systems, pollinator-mediated facilitation in plant communities is thought to occur through several mechanisms. First, coexisting plants may attract greater numbers of shared pollinators by providing aggregate floral displays greater than a single species alone^44^. Facilitation may also occur when species with staggered blooming periods support pollinator populations by reducing spatial and temporal variation in floral resource availability^49^. In this case, the consecutive bloom of plant species increases the duration of floral resource availability within years or the reliability of floral resources across years^50^. These same dynamics may be particularly important for pollinator communities in agricultural systems where crop rotation or extreme weather events can lead to high variability in floral resource abundance among seasons and years. If the greater abundance, diversity and duration of floral resources can be achieved through complementarity of flowering crops, later blooming crops such as strawberry may even support the pollination services of earlier mass flowering crops in the following year^51^.

In agricultural systems, our findings reveal that crop habitats can act as a source of ecosystem services to other crops and represent an area of underexploited potential for ecological intensification practices. Studies of spillover of pollinators between mass flowering crops have also reported that prevalence of early mass flowering crops in the landscape can mitigate pollinator dilution in another mass flowering crops blooming in a later season^21^. Our results advance these findings by demonstrating that changes in the abundance of pollinators mediated by the bloom of mass flowering crops has consequences for the yield of nearby pollinator dependent crops. Importantly, our results highlight the importance of timing in determining the outcome of interactions among pollinator dependent crops and suggest ecological intensification strategies that may be employed to reduce competition and enhance facilitation among crops that have a significant number of shared pollinators. By selecting crops and varieties that bloom sequentially with shared pollinator communities, growers can minimize competition while maximizing facilitative effects, thereby improving the sustainability of crop pollination. However, when agronomic or other factors constrain variety selection, management strategies may focus on locating co-blooming crops at distances greater than the average foraging range of their shared pollinator community.

## Conclusion

Our results clearly indicate that the timing of flowering in co-occurring crops can have consequences for the yield of pollinator dependent plants. When one crop co-blooms with another, mass flowering crop, competition for pollinators is likely to reduce yield, while flowering after the flowering event facilitates pollination leading to higher yields. We show that the temporal resource pulses associated with mass flowering crops are an important driver of pollinator community dynamics and pollination services at local and landscape scales. Greater understanding of these effects will allow for improvements in designing agroecosystems in order to maximize the provisioning of ecosystem services and promote crop yields through ecological intensification.

## Additional Information

**How to cite this article:** Grab, H. *et al*. Temporally dependent pollinator competition and facilitation with mass flowering crops affects yield in co-blooming crops. *Sci. Rep.* **7**, 45296; doi: 10.1038/srep45296 (2017).

**Publisher's note:** Springer Nature remains neutral with regard to jurisdictional claims in published maps and institutional affiliations.

## Supplementary Material

Supplementary Information

## Figures and Tables

**Figure 1:**
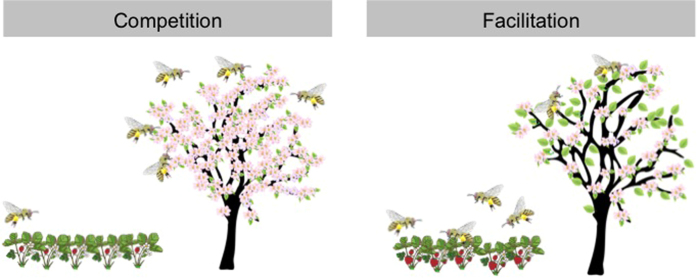
A simple conceptual model for the consequences of pollinator sharing between a mass flowering and co-blooming crop. (**A**) Pollinator spillover from co-blooming crops to mass flowering crops during mass flowering results in competition for pollinators and a decrease in co-blooming crop yields. (**B**) Pollinator spillover into co-blooming crops following bloom of mass flowering crops results in facilitation of pollinator visitation to co-blooming crops and an increase in co-blooming crop yields.

**Table 1:** Minimum adequate models describing local and landscape scale effects on abundance and species richness of bees in strawberry fields sampled during early, peak and late apple bloom from sites located adjacent or distant from natural areas.

Variable	df	*F*	*P*
Bee Abundance			
Stage	2,48	2.10	0.133
Natural Habitat Proximity	1,48	3.86	0.055
Mass Flowering Index	1,48	9.15	0.004
Stage X Index	2,48	8.41	0.001
Bee Species Richness			
Stage	2,49	0.94	0.394
Mass Flowering Index	1,49	2.14	0.149
Stage X Index	2,49	6.80	0.003

**Table 2:** ANOVA table output of minimum adequate models describing landscape scale effects of apple mass flowering on the weights of hand-pollinated and open-pollinated of sentinel strawberry fruits sampled during early, peak and late apple bloom.

Variable	df	*F*	*P*
Pollination Treatment	1,876	0.728	0.393
Flowering Stage	2,876	7.757	0.001
Mass Flowering Index	1,876	0.004	0.946
Poll. Trt. X Index	1,876	7.224	0.007
Stage X Index	2,876	8.322	0.001
