# Supplementary material for: Temporally dependent pollinator competition and facilitation with mass flowering crops affects yield in co-blooming crops
Source: Sci Rep. 2017 Mar 27;7:45296. doi: 10.1038/srep45296 (PMC5366890; doi:10.1038/srep45296)
Supplement: Supplementary Information [file srep45296-s1.pdf]

SUPPLEMENTARY INFORMATION

Temporally dependent pollinator competition and facilitation with mass flowering crops affects yield in co-blooming crops

Authors: Heather Grab[a,b,1], Eleanor J. Blitzer[c], Bryan Danforth[a], Greg Loeb[b], Katja Poveda[a]

[a] Department of Entomology, Cornell University, Ithaca, New York 14853, United States
[b] Department of Entomology, New York State Agricultural Experiment Station, Cornell University, Geneva, New York 14456, United States
[c] Department of Biology, Carroll College, 295 S. Harrison Helena, MT 56901, USA.

[1]To whom correspondence should be addressed. Email: hlc66@cornell.edu, Phone: 717-364-6198.

Figure S1. Rarefaction curve characterizing sampling coverage of the species richness for the regional species pool.

Figure S2. Rarefaction curve characterizing sampling coverage of the species richness for sampling with semi-natural habitats.

Figure S3. Rarefaction curve characterizing sampling coverage of the species richness for sampling within strawberry fields.

Figure S4. NMDS plots of bee communities collected in bee bowls near sentinel strawberry arrays during the early, peak and late stages of apple bloom. Community composition differed marginally between stages (permanova, $F_{(2,47)} = 1.23$, $p = 0.053$). Earlier stages comprised of a subset of the community present in the following stage. Differences in community composition between stages was assessed with a permutational manova (999 permutations) implemented with the adonis function in the R package picante.

Figure S5. NMDS plots of bee communities collected in bee bowls near sentinel strawberry arrays at locations within strawberry fields or adjacent to nearby natural habitats. Community composition differed between sites (permanova, $F_{(2,47)} = 2.35$, $p = 0.002$). Differences in community composition between stages were assessed with a permutational manova (999 permutations) implemented with the adonis function in the R package picante.

Table S1. ANOVA table outputs for separate minimum models describing local and landscape scale effects on abundance and species richness of bees in strawberry fields sampled during early, peak and late apple bloom from sites located adjacent or distant from natural areas.

Table S2. ANOVA table outputs of separate minimum adequate models describing landscape scale effects of apple mass flowering on the weights of hand-pollinated and open-pollinated of sentinel strawberry fruits sampled during early, peak and late apple bloom.

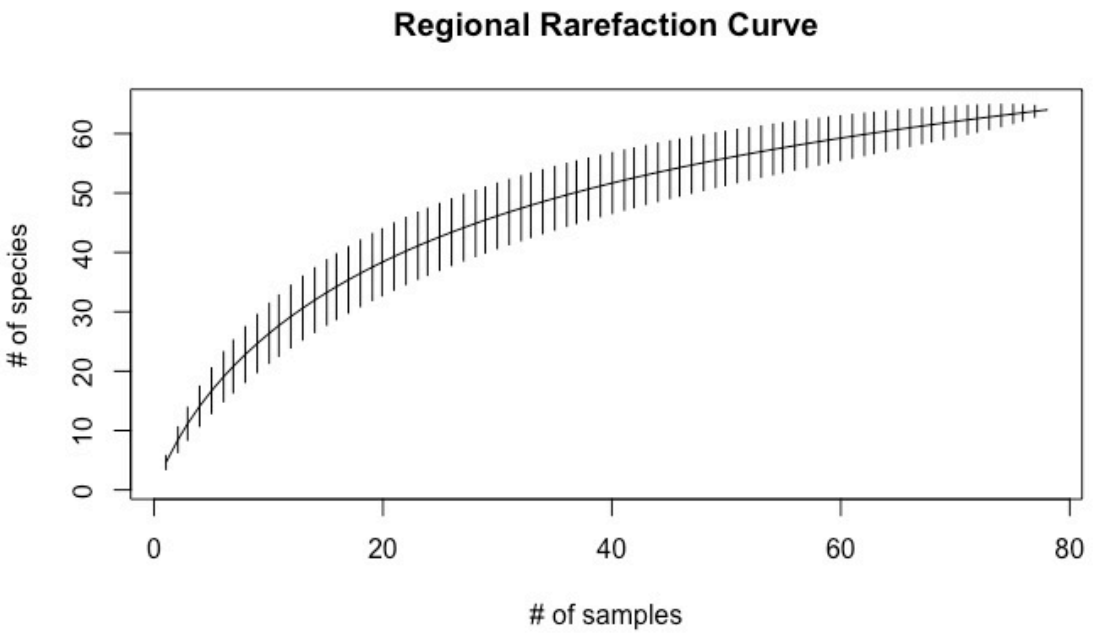

Regional Rarefaction Curve
# of species
0
10
20
30
40
50
60
0
20
40
60
80
# of samples

Figure S1.

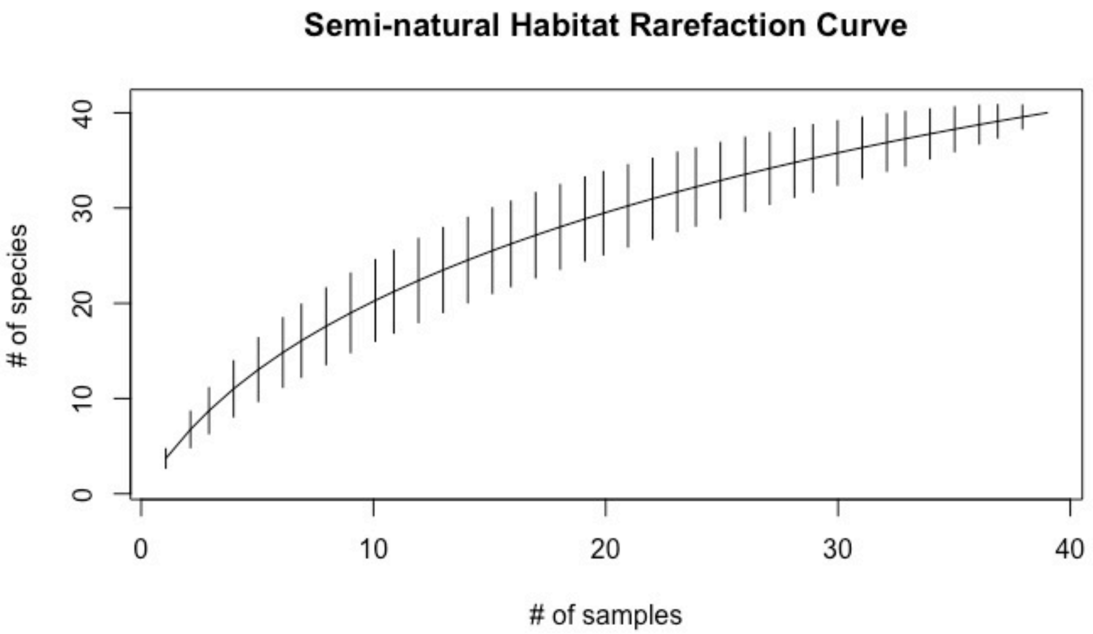

Semi-natural Habitat Rarefaction Curve
# of species
# of samples
0
10
20
30
40
0
10
20
30
40

Figure S2.

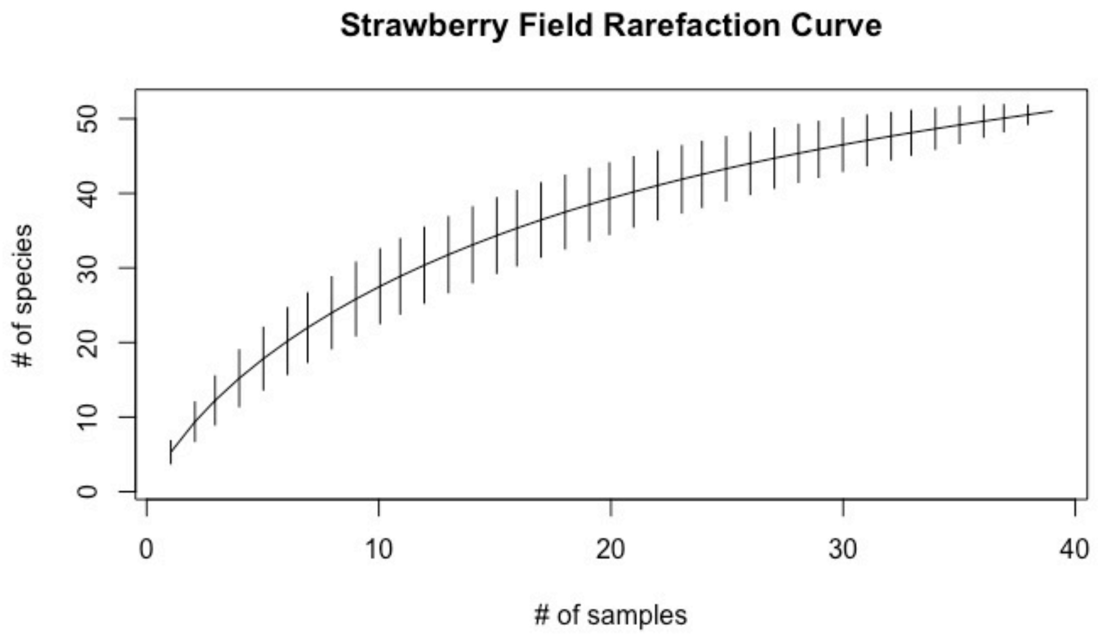
Strawberry Field Rarefaction Curve
# of species
0
10
20
30
40
50
0
10
20
30
40
# of samples

Figure S3.

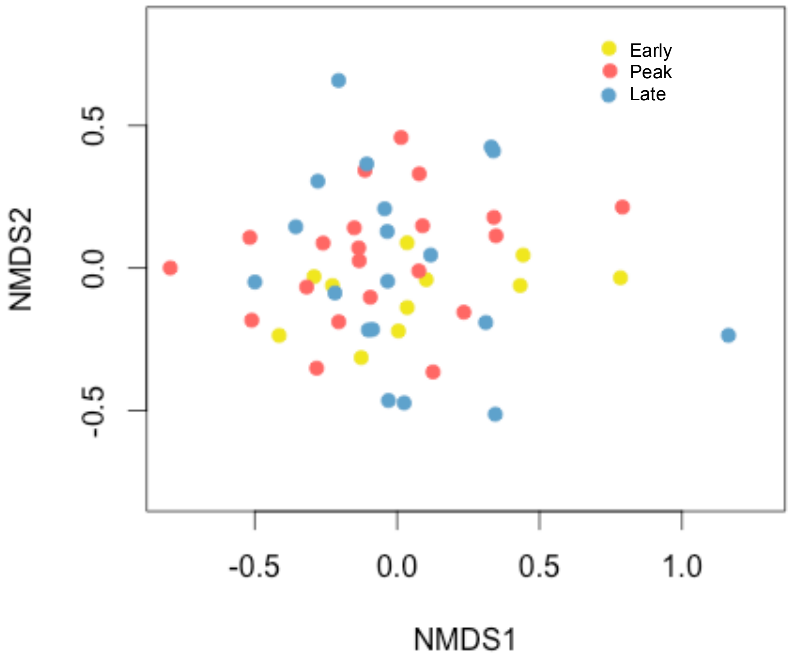

Figure S4.

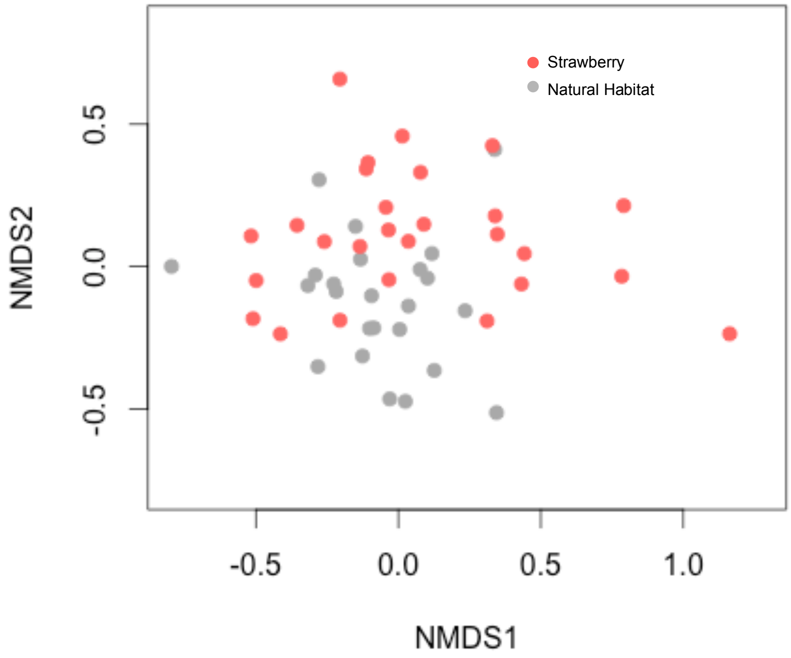

Figure S5.

Table S1.

Variable	df	*F*	*P*
Bee Abundance			
Early Apple Bloom			
Natural Habitat Proximity	1,6	0.71	0.429
Mass Flowering Index	1,6	5.51	0.057
Peak Apple Bloom			
Natural Habitat Proximity	1,13	2.40	0.145
Mass Flowering Index	1,13	11.38	0.005
Late Apple Bloom			
Natural Habitat Proximity	1,10	1.27	0.285
Mass Flowering Index	1,10	5.60	0.039
Bee Species Richness			
Early Apple Bloom			
Mass Flowering Index	1,6	9.66	0.021
Peak Apple Bloom			
Mass Flowering Index	1,14	7.33	0.017
Late Apple Bloom			
Mass Flowering Index	1,11	8.47	0.014

Table S2.

Variable	df	*F*	*P*
Early Apple Bloom			
Pollination Treatment	1,346	0.785	0.376
Mass Flowering Index	1,346	8.435	0.027
Poll. Trt. X Index	1,346	5.677	0.017
Peak Apple Bloom			
Pollination Treatment	1,408	5.012	0.002
Mass Flowering Index	1,408	1.500	0.221
Poll. Trt. X Index	1,408	4.519	0.034
Late Apple Bloom			
Pollination Treatment	1,172	0.001	0.978
Mass Flowering Index	1,172	4.069	0.045
Poll. Trt. X Index	1,172	0.424	0.515
